# Epicardial Fat in Heart Failure and Preserved Ejection Fraction: Novel Insights and Future Perspectives

**DOI:** 10.1007/s11897-025-00700-5

**Published:** 2025-03-19

**Authors:** Jacob Whitman, Elie Kozaily, Erin D. Michos, Daniel N. Silverman, Marat Fudim, Robert J. Mentz, Ryan J. Tedford, Vishal N. Rao

**Affiliations:** 1 Department of Medicine, Duke University School of Medicine, Durham, NC USA; 2 Division of Cardiology, Medical University of South Carolina, 30 Courtenay Drive, MSC Code: 592, Charleston, SC 29425 USA; 3 Division of Cardiology, Johns Hopkins University School of Medicine, Baltimore, MD USA; 4 Division of Cardiology, Ralph H. Johnson Department of Veterans Affairs Heath Care System, Charleston, SC USA; 5 Division of Cardiology and Duke Clinical Research Institute, Duke University School of Medicine, Durham, NC USA

**Keywords:** Obesity, Epicardial adipose tissue, Regional adiposity, Heart failure, HFpEF

## Abstract

**Purpose of Review:**

Cardiovascular effects of obesity may be driven, in part, by the distribution of fat. More recently, epicardial adipose tissue (EAT) has gained recognition as an adverse visceral fat impacting cardiac dysfunction in heart failure with preserved ejection fraction (HFpEF).

**Recent Findings:**

EAT can be identified and measured using several non-invasive imaging techniques, including transthoracic echocardiography, computed tomography, and cardiac magnetic resonance. The presence of EAT is associated with increased risk of HFpEF and worse clinical outcomes among patients with established HFpEF, independent of total adiposity. EAT may serve a pivotal role in the pathogenesis of HFpEF by worsening volume distribution, enhancing pericardial restraint and ventricular interaction, worsening right ventricular dysfunction, and diminishing exercise tolerance. No large trials have tested the effects of reducing fat in specific areas of the body on cardiovascular outcomes, but some studies that followed people in communities and trials over time have suggested that drug and non-drug treatments that lower EAT could improve the risk factors for heart problems in patients with HFpEF.

**Summary:**

Further understanding the role that pathogenic fat depots play in HFpEF incidence and progression may provide future therapeutic targets in treating the obese-HFpEF phenotype.

## Introduction

Heart failure (HF) with preserved ejection fraction (HFpEF) is a heterogeneous syndrome defined by abnormal left ventricular relaxation and filling with an ejection fraction ≥ 50% [1]. Its pathophysiology and optimal management remain complex given its rising incidence worldwide, constituting over half the population affected by HF [2–5]. Obesity (characterized by increased total adiposity) is a common comorbidity amongst HFpEF patients and is responsible for significant population attributable risk for new-onset HFpEF [6–8]. Additionally, a distinct obese-HFpEF pathophysiologic phenotype exists among patients with established HF, characterized by challenging volume assessment, enhanced pericardial restraint and ventricular interaction, worsening right ventricular dysfunction, and diminished exercise tolerance [9–11]. Due to the rising prevalence of comorbid obesity and HFpEF [1, 10], understanding pathophysiologic interactions between adiposity and HFpEF and potential therapeutic options are imperative to optimize patient care.

Obesity is most commonly defined by anthropometrics, such as body mass index. However, regional adiposity may contribute more to observed adverse cardiovascular effects than total adiposity alone [12]. Regional adiposity accumulates in visceral (intraabdominal and epicardial), subcutaneous, and intramuscular compartments. Visceral adipose tissue (VAT) exhibits biochemically unique properties with intrinsic endocrine function, and serves as a risk factor for cardiometabolic disorders and HFpEF [10, 13, 14].

Located between the myocardium and the epicardium, EAT is unique among visceral fat depots as it shares blood supply with the heart and directly interacts with myocardial structures [15, 16]. Epicardial adipose tissue (EAT) may have the greatest prognostic utility among visceral fat compartments. Higher EAT is associated with impaired cardiac function, elevated filling pressures, worsened pericardial restraint, decreased atrial and ventricular strain, and impaired exercise tolerance [17–21]. Notably, increased EAT is positively associated with both incident HFpEF and mortality after accounting for conventional cardiovascular risk [22]. Among patients with established HFpEF, EAT is associated with HF hospitalizations and mortality [17, 23, 24].

In this review, we describe the proposed mechanisms by which excess EAT mediates myocardial dysfunction, review clinical imaging techniques for measuring excess EAT, and explore treatment options that may potentially attenuate deleterious effects of EAT in HFpEF.

## Epicardial Adipose Tissue Quantification and Heart Failure with Preserved Ejection Fraction Associated Outcomes

The relationship between epicardial fat and clinical outcomes among populations at-risk for and with HFpEF have been increasingly evaluated. Among the large, community-based cohorts of the Multi-Ethnic Study of Atherosclerosis and Jackson Heart Study, pericardial fat volume (quantifying adjacent pericardial and epicardial adiposity) was associated with increased risk for incident HFpEF in both men and women [22, 25]. In patients with established HFpEF, both EAT thickness and volume are associated with increased likelihood of cardiovascular death and heart failure-related hospitalizations, even when controlling for total adiposity and other comorbidities [17, 24, 26]. Interestingly, lower EAT density by measure of fat attenuation index (FAI) is also associated with increased rates of mortality and heart failure hospitalization [27].

An ongoing area of investigation entails the relationship between sex and EAT in patients with HFpEF. Multiple studies suggest that female sex is associated with greater quantified EAT in patients with HFpEF [19, 24]. Higher observed EAT has also been associated with greater degree of pericardial restraint, particularly among women [28]. Furthermore, female sex is associated with increased odds for worsening exercise capacity in HFpEF [29, 30]. However, other groups have found contrasting results, in which male sex is associated with increased EAT in HFpEF [31], and the association between EAT and cardiovascular outcomes among patients with HFpEF are not mediated by sex [26]. These studies to date indicate greater attention is needed in systematically quantifying EAT across sexes in order to better understand the role of EAT on cardiac dysfunction and clinical outcomes in HFpEF.

An important and growing area of research focuses on changes in EAT and associated HFpEF risk. Retrospective cohort studies have shown an associated increased risk for incident HFpEF by baseline and interval changes in pericoronary pericardial adipose tissue and EAT thickness [25, 32]. Prospective data are needed to further clarify the associations between regional EAT, interval changes in EAT, and HFpEF outcomes.

## Pathophysiology of EAT-Mediated Dysfunction in HFpEF

In the healthy heart, EAT is principally located around the coronary arteries, interventricular and atrioventricular grooves, atria, right ventricle, and apex [15]. EAT is anatomically contiguous with the underlying myocardium and shares a blood supply, allowing for paracrine signaling [15]. Primarily consisting of adipocytes, EAT also includes macrophages, lymphocytes, stromovascular cells, and nerve ganglia [33]. Physiologically, it serves as a buffer between the myocardium and excess peripheral fatty acids, reduces mechanical stress on the heart, functions as storage of macromolecules such as free fatty acids and adiponectin, and additionally plays a role in thermogenesis [34].

**Fig. 1:**
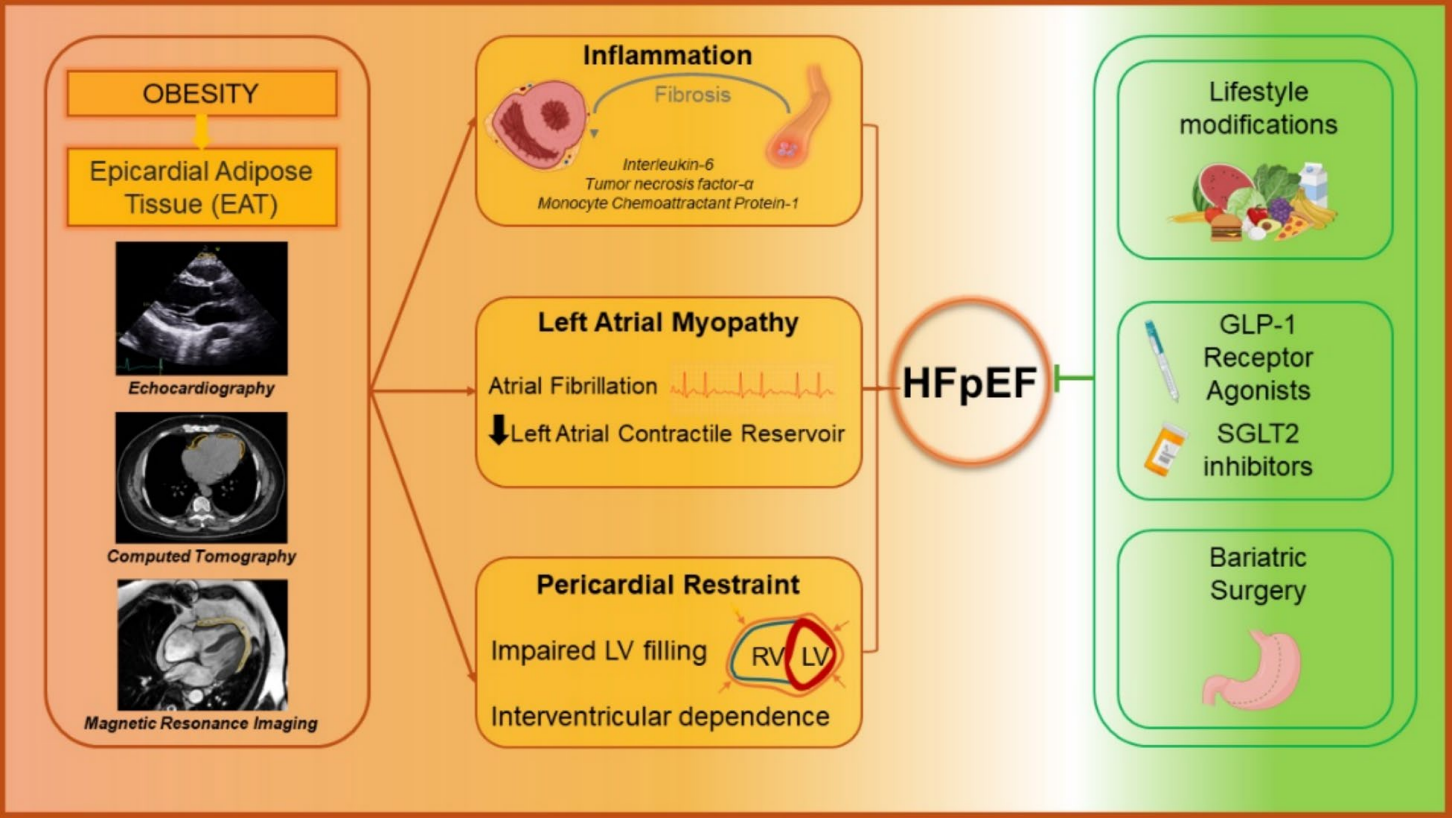
Pathophysiologic mechanisms by which epicardial adipose tissue mediates myocardial dysfunction in heart failure with preserved ejection fraction. Abbreviations: GLP-1, glucagon-like peptide-1; HFpEF, heart failure with preserved ejection fraction; LV, left ventricular; RV, right ventricular; SGLT2; sodium-glucose cotransporter 2

Several hypotheses have been proposed to describe the effects of increasing EAT on HFpEF myocardial dysregulation. One is the “infiltrative lipotoxicity” model, which centers on paracrine activity and myocardial infiltration by EAT. A second is the “pericardial restraint” model, which describes mechanistic impairment of myocardial function through EAT expansion within fixed pericardial space. A third proposed mechanism is alterations in left atrial function by EAT with resultant atrial arrhythmias that worsen cardiac function in HFpEF. While each model is presented separately in this review, multiple mechanisms may serve significant roles in mediating the pathophysiologic derangements in HFpEF (Fig. 1).

### Infiltrative Lipotoxicity Model

In healthy individuals, EAT serves as a direct mediator for paracrine signaling by releasing anti-inflammatory cytokines such as adiponectin [35]. With EAT expansion, its secretome profile changes, releasing more pro-inflammatory and pro-fibrotic cytokines such as interleukin-6 (IL-6), tumor necrosis factor-alpha (TNF-a), and monocyte chemoattractant protein-1 (MCP1) [35]. The upregulation of pro-inflammatory and pro-fibrotic factors leads an oxidative stress response, change in lipid metabolism, mitochondrial dysfunction, collagen metabolism, and ultimately cardiac fibrosis [36–39]. In addition to its paracrine and vasocrine mediated effects, EAT may also infiltrate the myocardium directly, resulting in changes in cardiomyocyte triglyceride content [39]. These observed changes have been associated with impaired diastolic dysfunction independent of age, body mass index (total adiposity), and other comorbidities seen in HFpEF [39]. Finally, ribonucleic acid (RNA) sequencing analyses of EAT derived from patients with HFpEF have revealed differential expression of non-coding RNAs involved in inflammation, mitochondrial regulation, cell death, fibrosis, and primary metabolic processes when compared to individual controls without HF [40, 41]. The significance of non-coding RNAs in EAT-mediated cardiac dysfunction is relatively understudied, and further research may reveal novel targets for therapeutic intervention.

### Pericardial Restraint Model

EAT resides within an enclosed space between the myocardium and visceral pericardium. The accumulation of EAT has been shown to impair ventricular strain and contractility at rest and with exercise across populations with obesity, diabetes, cardiovascular diseases, and established HFpEF [17, 42–46]. EAT accumulation is associated with impaired ventricular diastolic filling [17, 20]. Among patients with HFpEF undergoing simultaneous echocardiography and invasive cardiac catheterization, higher EAT thickness is associated with hemodynamic patterns of pericardial restraint, as demonstrated by the presence of a “square-root sign,” and correlated with impaired ventricular filling, enhanced ventricular interaction, and reduced cardiac output [28]. As a consequence of impaired ventricular filling and cardiac output, it is postulated that increased sympathetic tone, volume redistribution, and venous return during exercise may further exaggerate pericardial restraint in HFpEF [10, 47]. The end-result may explain the abnormal changes observed with elevated filling pressures, worsened peak oxygen consumption and peripheral oxygen extraction that culminate in exercise tolerance seen in patients with HFpEF [17, 19, 20].

### Epicardial Adipose Tissue and Atrial Fibrillation

Atrial fibrillation (AF) is common in patients with HFpEF and is associated with worse clinical outcomes when presenting comorbidly [48]. The presence of EAT is associated with impaired left atrial contractile reserve across populations with and without established HF [17, 18, 43, 44, 49]. Among patients with comorbid HFpEF and AF, left atrial compliance is reduced and associated with increased pulmonary congestion and diminished exercise capacity [50]. AF may also be associated with mitral and tricuspid valve regurgitation, leading to further pulmonary congestion and impaired cardiac output [51, 52]. As a result, higher EAT in HFpEF may augment the burden of AF [53, 54], possibly through aberrations in atrial conduction resulting from paracrine-signaling, NLRP3 inflammasome activity, and adipose infiltration into atrial myocardium [55–58]. These observed interactions between EAT and atrial function in HFpEF may characterize a distinct pathophysiologic mechanism and potential therapeutic target.

## Imaging Techniques of Epicardial Adipose Tissue

**Fig. 2:**
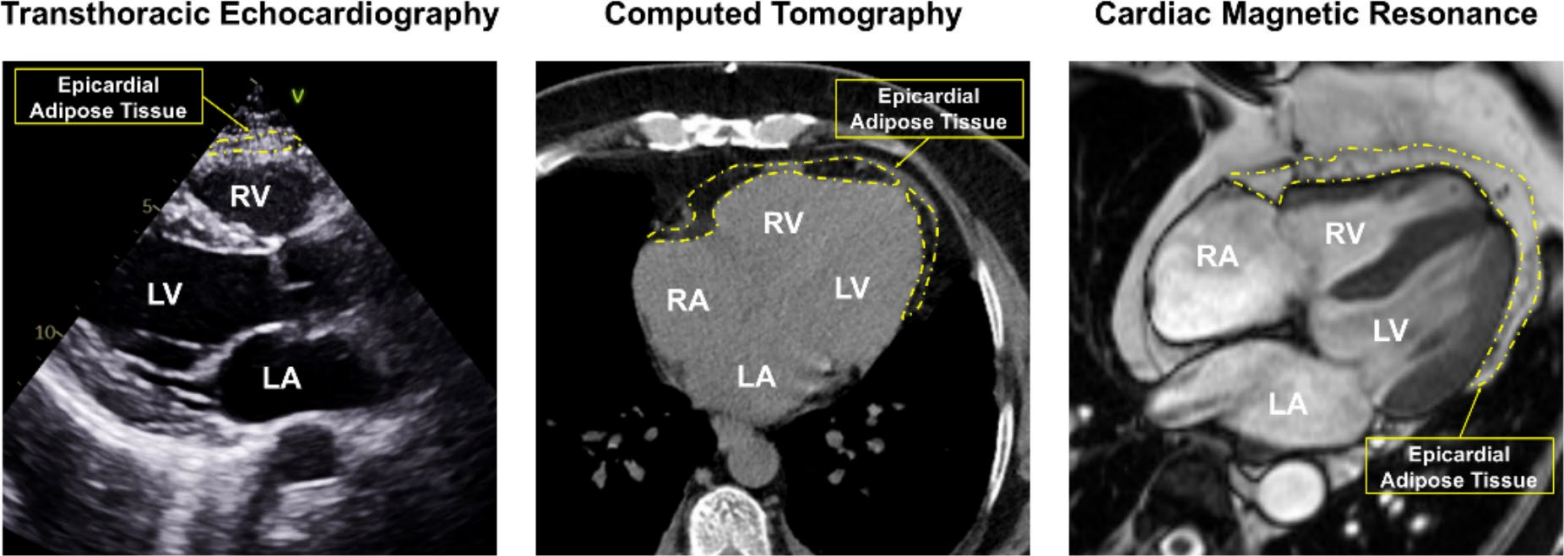
Cardiovascular imaging techniques for measuring epicardial adipose tissue thickness and volume. Epicardial adipose tissue (EAT) can be measured as thickness using transthoracic echocardiography (left), or as single-slice areas or multi-slice volumes using computed tomography (middle) and cardiac magnetic resonance (right) imaging techniques. EAT is displayed within yellow borders. *Abbreviations: RA, right atrium; RV, right ventricle; LA, left atrium; LV, left ventricle*

Multiple imaging modalities currently used in clinical practice have demonstrated feasibility in measuring EAT. These include transthoracic echocardiography (TTE), computed tomography (CT), and cardiac magnetic resonance (CMR) imaging. Each of these clinical imaging modalities exhibit varying strengths and limitations in quantifying EAT and may inform future clinical applicability (Table 1, Fig. 2).

**Table 1:** Available imaging techniques to quantify epicardial adipose tissue

Two-Dimensional Transthoracic Echocardiography (TTE)		Cardiac Magnetic Resonance (CMR) and Computed Tomography (CT) imaging	
EAT thickness is defined as the hypoechoic space between the visceral pericardium and outer myocardium along the free wall of the right ventricle in parasternal-long axis view		CT: EAT is delineated as epicardial tissue between −250 and −30 Hounsfield units in each short-axis slice with total volume calculated using modified Simpson’s rule CMR: EAT delineated manually and summed in a similar fashion to calculate total EAT volume	
Pros: • Most time and cost-efficient imaging modality. Ideal for widespread screening • Reasonably good inter-observer and intra-observer reliability (Parisi 2020) • Thickness is well-correlated with volume as measured by three-dimensional techniques (Parisi 2020) • Most widely used imaging modality for EAT research • Measurement protocol is relatively standardized throughout multiple studies	Cons: • Does not allow for volumetric assessment of EAT • Cannot assess regional variations in EAT • Quality of the obtained images is often limited by the patient’s body habitus • Not as useful for measuring inflammation, density, white/brown content, and other characteristics of EAT	Pros: • Higher spatial resolution than 2D imaging modalities • Can assess total EAT volume and regional variations in EAT • CT fat attenuation index can be used to assess EAT inflammation (Liu 2023) • CMR can be used to assess EAT infiltration into the myocardium (Wu et al. 2020) and assess the effects of local variations in EAT on underlying function (Van Woereden 2021) • Brown adipose tissue content can be assessed using FDG-PET techniques (Ouwerkerk 2021)	Cons: • Costly, labor intensive, and less time efficient than 2D measurement methods • Measurement protocols are not as widely standardized as 2D imaging modalities • Use of 3D imaging techniques in research studies has been relatively limited • Correlation of EAT volume with cardiac function and patient outcomes is not as well-validated

### Transthoracic Echocardiography

TTE is the most commonly available modality for EAT assessment due to lower cost, ease of use, and reproducibility [15]. EAT presents as an anechoic space located between the myocardium posteriorly and the visceral pericardium anteriorly, though it can occasionally have increased echogenicity at greater quantities or in the presence of inflammation [59]. Two-dimensional EAT thickness can be measured along the right ventricular free wall at end-systole. [59]. EAT thickness as measured by TTE correlates well with three-dimensional volumetric measures of EAT. The inter-observer and intra-observer reliability of TTE, though inferior to CT and MRI, makes it appropriate tool for EAT quantification in studies of EAT [60, 61].

### Computed Tomography and Cardiac Magnetic Resonance Imaging

CT and CMR provide three-dimensional assessment of EAT, often using a “slice-by-slice” approach wherein EAT is manually delineated across cross-sectional slices that are totaled using the modified Simpson’s rule to attain a volume measure [31, 42, 61]. As opposed to the single-slice method utilized in TTE, three-dimensional imaging allows for the assessment and quantification of subtle regional variations in EAT. However, CT/CMR-derived EAT volumes can be labor-intensive and time-consuming, and their accessibility and cost may limit widespread availability for routine quantification of EAT volumes. Alternatively, single-slice approaches have been utilized, although, like TTE, these cannot account for varying myocardial distribution of EAT that may limit subtle changes in total EAT volume [62]. Several investigators have explored ways in which artificial intelligence and machine-based learning may be applied to expedite the process of image acquisition and segmentation [63, 64] and EAT volumetric quantification [65] with promising preliminary results. Thus, the application of image analysis algorithms may improve volumetric assessments of EAT in order to provide greater opportunities to characterize regional adiposity and cardiovascular outcomes, as has been shown with the application of machine learning-derived risk score inclusive of traditional risk factors and coronary artery calcium scoring [66].

### Novel Methods to Quantify EAT

While previous studies have explored EAT thickness and volumes, others have explored unique features of EAT through novel imaging techniques. FAI, as measured by CT Hounsfield units, describes the relative lipid content and adipocyte size within EAT volumes and has been used to evaluate inflammation, fibrosis, and overall fat density [27]. Among patients with established HFpEF, FAI may provide greater prognostic value for cardiovascular risk than EAT volume [27]. This low-density EAT is postulated to be associated with increased adipocyte hypertrophy, proliferation, fibrosis, inflammation, and increasing white adipose tissue content [27]. Additionally, brown-to-white fat ratio within EAT as measured by proton magnetic resonance spectroscopy, may also serve as a useful tool and has demonstrated associations between brown adipose tissue and cardiac function [67].

The myocardial regional variation of EAT may also confer differences in cardiac structure and function. EAT has previously been shown to exhibit differential protein expression depending upon its location along the myocardium, suggesting the possibility for activation and suppression of different cytokine and metabolic pathways when under pathologic stress [68]. Quantified higher regional variation in EAT among patients with established HFpEF is associated with greater left ventricular mass and volume and worse diastolic dysfunction when EAT directly overlies the left ventricle, even after adjusting for total EAT [31]. Similarly, deficits in right ventricular longitudinal strain with exercise correlate with higher EAT overlying the right ventricle [69]. With continued improvements in imaging algorithms to quantify EAT, future studies may better characterize the significance of EAT density and regional distribution and associated pathophysiology of HFpEF.

## Epicardial Adipose Tissue Directed Therapies

While the vast majority of therapeutic interventions to date have focused on total weight or adiposity, several pharmacologic and non-pharmacologic interventions have demonstrated improvements in EAT with variable changes in cardiac function (Table 2). The following section discusses existing and ongoing research of therapies provided for obesity and effects on EAT modulation. These interventions include lifestyle modifications, pharmacotherapies including glucagon-like peptide-1 receptor agonists (GLP-1a) and sodium-glucose cotransporter-2 inhibitors (SGLT2i), and bariatric surgery. In addition to their effects on EAT and other VAT depots, most of the therapies discussed below also have a reducing effect on total body adiposity. Outside of direct surgical resection, there are no currently available therapies that target EAT alone. While not the primary focus of this review, it should be noted that several other drug classes have demonstrated potential efficacy for reducing EAT, including HMG-CoA reductase inhibitors [70–73], biguanides [74], and proprotein convertase subtilisin/kexin type 9 inhibitors [75].

**Table 2:** Available data from randomized clinical trials examining associated changes in total adiposity and EAT by SGLT2i or GLP-1a

Randomized trial (Year)*	Treatment groups (*N*)	Patient characteristics	Trial duration	Adiposity-related endpoint(s)	Results
Kosiborod et al. (2023)	Semaglutide (263) vs. Placebo (266)	Patients with HFpEF and BMI ≥ 30	52 weeks	Mean percent change in total body weight from baseline	Semaglutide −13.3%, Placebo −2.6%, estimated difference 10.7% (*p* < 0.01)
Lincoff et al. (2023)	Semaglutide (8803) vs. Placebo (8801)	Patients without diabetes and established CVD with BMI ≥ 27	104 weeks	Mean percent change in total body weight	Semaglutide −9.39%, Placebo −0.88%, estimated difference −8.51% (CI −8.75 to −8.27)
Iacobellis et al. (2020)	Semaglutide (30) vs. Dulaglutide (30) vs. Metformin (20)	Patients with type 2 diabetes and BMI ≥ 27	12 weeks	Change in EAT thickness from baseline	Significant decrease in EAT thickness in semaglutide and liraglutide groups (~ 20% reduction in both groups). No significant EAT reduction seen in metformin group
Iacobellis et al. (2017)	Liraglutide + Metformin vs. Metformin alone	Patients with type 2 diabetes and BMI ≥ 27	3 and 6 months	Change in EAT thickness from baseline (mm)	Significant decrease in EAT in semaglutide group (9.6 ± 2 to 6.8 ± 1.5 and 6.2 ± 1.5 at 3 and 6 months respectively, *p* < 0.001). No significant change seen with metformin monotherapy
Sato et al. (2018)	Dapagliflozin (20) vs. Non-SGLT2i oral diabetes medications (20)	Patients with diabetes (A1c ≥ 6.5%) and coronary artery disease	6 months	Change in EAT volume from baseline (cm^3^)	Significant decrease in EAT volume in dapagliflozin group (− 16.4 ± 8.3 vs. 4.7 ± 8.8, *p* = 0.01)
Iacobellis et al. (2020)	Dapagliflozin + Metformin (50) vs. Placebo + Metformin (50)	Patients with type 2 diabetes with BMI ≥ 27 and A1c ≤ 8 on metformin monotherapy	24 weeks	Change in EAT thickness from baseline	Dapagliflozin: 20% reduction from baseline Placebo: 7% reduction from baseline

^*^To date, there have been no trials examining direct changes to EAT by SGLT2i or GLP-1a in patients with established HFpEF

Abbreviations: *BMI* Body mass index; *EAT* Epicardial adipose tissue; *GLP1a* Glucagon-like peptide-1 receptor agonists; *HFpEF* Heart failure with preserved ejection fraction; *SGLT2i* Sodium-glucose cotransporter-2 inhibitor

### Lifestyle Modifications

Effective dietary and lifestyle counseling is paramount to the appropriate management of the obesity-HFpEF phenotype. Obesity is associated with declining exercise tolerance in patients with HFpEF [9]. Weight loss is associated with improved oxygen consumption and cardiac hemodynamics at rest and with exertion [76]. The perceived benefits of lifestyle modifications in patients with HFpEF may in part be mediated by reductions in VAT depots, particularly EAT, despite total weight changes. The SECRET trial examined exercise tolerance among elderly patients with HFpEF and found that caloric restriction or aerobic exercise training increased peak oxygen consumption with potential additive effects [77]. Dieting yielded modest weight loss, although the investigators observed reduction in CMR-measured abdominal visceral and subcutaneous adiposity, but not epicardial/pericardial adiposity, and no changes in cardiac function [77]. Whereas, the CENTRAL trial, which examined isocaloric low-fat or Mediterranean/low-carbohydrate with and without exercise among sedentary obese adults, found CMR-derived pericardial fat decreased over 18 months independent of total observed weight loss [78].

While available dietary interventions are numerous, low carbohydrate, Mediterranean, and calorie-restricted diets have demonstrated efficacy in reducing EAT among patients with cardiovascular disease [78, 79]. Additionally, physical activity interventions including both endurance and resistance training have been shown to effectively reduce EAT [79]. Recent meta-analyses of multiple weight loss interventions on regional adiposity suggest that exercise training may selectively decrease EAT compared with dietary interventions and bariatric surgery [79]. Additionally, exercise-mediated EAT reduction may occur in the absence of total weight changes [80, 81]. A proposed mechanism of exercise benefits may be attributed to increased levels of IL-6, which may promotes lipolysis and EAT reduction [82]. However, cumulative data to date suggest that moderate physical activity alone may not significantly impact EAT, whereas when conducted alongside dietary interventions, these lifestyle modifications may confer greatest reduction in EAT beyond total adiposity changes [78]. As such, recommendations for diet and exercise should be mainstay in the treatment of obese-HFpEF, although future studies should further investigate how observed changes in EAT may improve cardiac function, exercise tolerance, and clinical outcomes in HFpEF [77, 78].

### Glucagon-Like Peptide-1 Receptor Agonists

Incretin-based treatments such as GLP-1a are approved for the treatment of type 2 diabetes and obesity and associated cardiovascular outcomes [83–86]. The recent STEP-HFpEF trial examined the use of semaglutide in patients with HFpEF and obesity and found large reductions in symptom burden and physical limitations, greater improvements in exercise capacity, and total weight loss. Interestingly, symptom improvement with GLP-1a use seemed to be independent of total weight loss, and patients with more severe disease obtained greater symptom improvement than patients with milder disease, despite similar weight loss across the spectrum of HFpEF severity [86]. In addition to their well-substantiated effects on total adiposity, GLP-1a are associated with significant reductions in all pathogenic visceral fat depots, including EAT [87]. Several studies have observed reductions in EAT thickness and volume with GLP-1a use [88–93], which appears to be dose-dependent [91] and regardless of glycemic control and total weight loss [88, 90, 91].

The mechanisms by which GLP-1a may preferentially improve visceral adiposity, including EAT, remain unclear. EAT expresses both GLP-1/2 receptors more abundantly than subcutaneous adipose tissue. potentially explaining the disproportionate effect of GLP-1 agonists on EAT reduction compared with other regional adiposity [94]. Furthermore, EAT GLP-1 receptor expression correlates with upregulation of genes involved with a shift in fatty acid metabolism from adipogenesis towards beta-oxidation, in addition to improved brown-to-white fat differentiation [95]. The promotion of a brown fat phenotype within the EAT (i.e., browning) has been suggested as a therapeutic strategy for mitigating EAT-associated myocardial dysfunction, since EAT is predominantly composed of brown adipose during childhood and gains properties of white adipose tissue with increasing age and total adiposity. Promotion of EAT browning, via pharmaceuticals and lifestyle changes, is postulated to reduce inflammation, improve glucose and fatty acid metabolism, and promote thermogenesis within the EAT, with potential associated benefits on myocardial function [34, 96]. The association of GLP-1 receptor expression with the expression of genes involved with brown-to-white adipose differentiation suggests that GLP-1a may mediate their therapeutic effects via EAT browning. Along with their beneficial effects on fatty acid metabolism, GLP-1a may also reduce EAT-associated pro-inflammatory signaling through modulation of exosome protein expression [97].

In addition to expressing GLP receptors, EAT has also been shown to express receptors of both gastric inhibitory polypeptide and glucagon, the expression levels of which are associated with genes related to beta oxidation, adipogenesis, and white-to-brown adipocyte differentiation [98]. This suggests a potential role for dual agonists GLP1/GIP and more recently developed GLP/GIP/GLUC triagonists in reducing and modulating the deleterious effects of EAT.

### Sodium-Glucose Cotransporter-2 Inhibitors

The effects of SGLT2i on clinical outcomes, including improved mortality and reduced heart failure-related hospitalization, in patients with HFpEF are well-established [99, 100]. The mechanisms of this benefit likely extend beyond their glucose-lowering effect and include reduction in inflammation and oxidative stress, which leads to reversal of cardiac remodeling and improved diastolic function [101]. SGLT2 is expressed by both mature adipocytes and pre-adipocytes in EAT and its expression is not related to indices of glycemic control [102, 103]. SGLT2i use is associated with a EAT reduction [104–112], and these observations appear independent of change in total body weight [109, 112] or total visceral adiposity [107]. EAT reduction may occur by SGLT2i-associated attenuation of systemic levels of inflammatory markers, including TNF-α [112], IL-6 [102], and C-reactive protein [106], suggesting a modulatory effect of SGLT2 inhibition on the pro-inflammatory profile of the EAT. However, prospective studies have yet to confirm SGLT2i on EAT changes in HFpEF, and whether reduction in EAT may improve cardiac function and fitness and long-term outcomes.

Mechanisms by which SGLT2i reduce EAT and myocardial dysfunction are also unclear. Some have postulated adipokine modulation, such as leptin downregulation and adiponectin upregulation, resulting in reduced systemic inflammation and cardiac fibrosis, improved insulin sensitivity, and enhanced antioxidant activity [113, 114]. Dapagliflozin is associated with EAT glucose uptake and utilization, which may mediate effects on insulin sensitivity [103]. Heightened insulin sensitivity leads to improved myocardial glucose metabolism and fatty acid utilization with a resultant decrease in EAT volume [15, 103]. However, the use of the euglycemic insulin-clamp method suggests SGLT2i-EAT interplay cannot completely explained by their effects on insulin sensitivity, perhaps indicating more complex mechanisms exist [110]. A recent pre-clinical study suggests that the effects of SGLT2i on cardiomyocyte mitochondrial function; including improved biogenesis, reduction in reactive oxygen species production, and optimized calcium distribution; are associated with normalization of the EAT secretome [115]. Importantly, EAT also expresses multiple other SGLT isoforms, including SGLT1 [102], and the combined effect of SGLT1/2 inhibitors in HFpEF patients is under investigation [116]. Future research may better characterize SGLT inhibitor class agents on various mechanism on regional adiposity and the interplay in HFpEF.

### Surgical Approaches to EAT Reduction

Bariatric surgery and weight loss is associated with improved TTE-measured parameters of diastolic dysfunction in HF [117]. However, the effects of bariatric surgery on long-term clinical outcomes in HFpEF remain understudied [118]. With regards to regional adiposity, bariatric surgery is associated with significant and maintained reductions in EAT throughout long-term follow-up [119–121].

Direct management of mechanical interaction between EAT and HFpEF has been explored via pericardiectomy. Pericardiectomy involves surgical excision of the pericardium adjacent to epicardial fat, potentially relieving a syndrome of impaired cardiac relaxation and enhanced interventricular dependence within a constrained space [122]. However, large randomized clinical trials of this therapy do not yet exist, and pericardiectomy provides inherent surgical risk with potential to adversely affect the important role of the pericardium on longitudinal right ventricular function [122]. Minimally invasive subxiphoid approaches to resect the anterior pericardium have been investigated in animal models and humans with improved early changes in filling pressures and ventricular volumes [123, 124], yet additional studies are warranted. To date, no other available clinical interventions exist that directly affect the role of EAT on cardiac function.

## Conclusion

The role of regional adiposity, particularly EAT, in the pathogenesis of HFpEF has gained increasing attention. EAT is a strong predictor for new-onset HFpEF, and its presence and epicardial distribution promotes pro-inflammatory myocardial dysfunction, enhanced pericardial restraint, and atrial arrhythmias, which later lead to myocardial fibrosis, impaired energy utilization, elevated filling pressures, and reduced exercise tolerance in HFpEF. Several pharmaceutical classes proven to improve clinical outcomes in HFpEF may also preferentially reduce EAT, highlighting an important role in the pathogenesis of HFpEF and its value as a potential therapeutic target. More research is necessary to further clarify whether EAT is a primary mediator or bystander in the obese-HFpEF phenotype. As clinical imaging techniques to identify and characterize EAT improve, future clinical trials may focus on evaluating EAT-modulating therapies across HFpEF populations.

## Key References

- Akoumianakis I, Zagaliotis A, Konstantaraki M, Filippatos TD. GLP-1 analogs and regional adiposity: A systematic review and meta-analysis. John Wiley and Sons Inc, 2023.
  - A metanalysis demonstrating the ameliorating effect of GLP-1 agonists on pathogenic visceral fat depots, including epicardial adipose tissue.
- Koepp KE, Obokata M, Reddy YNV, Olson TP, Borlaug BA. Hemodynamic and Functional Impact of Epicardial Adipose Tissue in Heart Failure With Preserved Ejection Fraction. Elsevier Inc., 2020:657–666.
  - A cross-sectional study, which evaluates the effects of increased epicardial adipose tissue on multiple indices of cardiac function and exercise tolerance.
- Rao VN, Fudim M, Mentz RJ, Michos ED, Felker GM. Regional adiposity and heart failure with preserved ejection fraction. John Wiley and Sons Ltd, 2020:1540–1550.
  - Review the article outlining the relationship between multiple adipose tissue depots and HFpEF.
- Van Woerden G, Van Veldhuisen DJ, Manintveld OC et al. Epicardial Adipose Tissue and Outcome in Heart Failure with Mid-Range and Preserved Ejection Fraction. Lippincott Williams and Wilkins, 2022:E009238.
  - A prospective study demonstrating the association between increasing epicardial adipose tissues and worsening clinical outcomes in patients with HFpEF.

## Data Availability

No datasets were generated or analysed during the current study.
